# Performance of the Universal Vital Assessment (UVA) mortality risk score in hospitalized adults with infection in Rwanda: A retrospective external validation study

**DOI:** 10.1371/journal.pone.0265713

**Published:** 2022-03-23

**Authors:** Riley Hazard, Danstan Bagenda, Andrew J. Patterson, Julia T. Hoffman, Steven J. Lisco, Olivier Urayeneza, Polyphile Ntihinyurwa, Christopher C. Moore

**Affiliations:** 1 University of Melbourne, School of Population and Global Health, Melbourne, Australia; 2 Department of Anesthesiology, University of Nebraska Medical Center, Omaha, NE, United States of America; 3 Department of Anesthesiology, Emory University, Atlanta, GA, United States of America; 4 University of Gitwe, School of Medicine, Gitwe, Rwanda; 5 Department of Surgery, California Hospital Medical Center, Los Angeles, CA, United States of America; 6 Department of Obstetrics and Gynecology, University of Rwanda, Kigali, Rwanda; 7 Division of Infectious Diseases and International Health, Department of Medicine, University of Virginia, Charlottesville, VA, United States of America; Fundacao Oswaldo Cruz, BRAZIL

## Abstract

**Background:**

We previously derived a Universal Vital Assessment (UVA) score to better risk-stratify hospitalized patients in sub-Saharan Africa, including those with infection. Here, we aimed to externally validate the performance of the UVA score using previously collected data from patients hospitalized with acute infection in Rwanda.

**Methods:**

We performed a secondary analysis of data collected from adults ≥18 years with acute infection admitted to Gitwe District Hospital in Rwanda from 2016 until 2017. We calculated the UVA score from the time of admission and at 72 hours after admission. We also calculated quick sepsis-related organ failure assessment (qSOFA) and modified early warning scores (MEWS). We calculated amalgamated qSOFA scores by inserting UVA cut-offs into the qSOFA score, and modified UVA scores by removing the HIV criterion. The performance of each score determined by the area under the receiver operator characteristic curve (AUC) was the primary outcome measure.

**Results:**

We included 573 hospitalized adult patients with acute infection of whom 40 (7%) died in-hospital. The admission AUCs (95% confidence interval [CI]) for the prediction of mortality by the scores were: UVA, 0.77 (0.68–0.85); modified UVA, 0.77 (0.68–0.85); qSOFA, 0.66 (0.56–0.75), amalgamated qSOFA, 0.71 (0.61–0.80); and MEWS, 0.74 (0.64, 0.83). The positive predictive values (95% CI) of the scores at commonly used cut-offs were: UVA >4, 0.35 (0.15–0.59); modified UVA >4, 0.35 (0.15–0.59); qSOFA >1, 0.14 (0.07–0.24); amalgamated qSOFA >1, 0.44 (0.20–0.70); and MEWS >5, 0.14 (0.08–0.22). The 72 hour (N = 236) AUC (95% CI) for the prediction of mortality by UVA was 0.59 (0.43–0.74). The Chi-Square test for linear trend did not identify an association between mortality and delta UVA score at 72 hours (p = 0.82).

**Conclusions:**

The admission UVA score and amalgamated qSOFA score had good predictive ability for mortality in adult patients admitted to hospital with acute infection in Rwanda. The UVA score could be used to assist with triage decisions and clinical interventions, for baseline risk stratification in clinical studies, and in a clinical definition of sepsis in Africa.

## Introduction

Sepsis is defined as life-threatening organ dysfunction caused by a dysregulated host response to infection and is the leading cause of mortality in low- and middle-income countries (LMICs) [1]. Sepsis definitions have changed from being based on consensus opinion to being based on patient derived models of increased risk of mortality due to an acute infection in patients from high income countries (HICs) [2]. Accordingly, the Sepsis-3 operational definition of sepsis requires an increase in sepsis-related organ failure assessment (SOFA) score of 2 points from baseline. A quick SOFA (qSOFA) score was also derived to identify patients at risk for increased mortality in the setting of infection where the components of the full SOFA score are not available. The qSOFA score has subsequently been retrospectively evaluated in patients from LMICs and found to provide additional information about mortality risk from baseline data alone [3].

Other studies from sub-Saharan Africa have separately evaluated qSOFA and found that it had variable performance depending on the population studied [4–6]. Because of the many differences in critically ill populations in LMICs compared to those in HICs, we recently used a large pooled dataset of hospitalized patients from 6 countries in sub-Saharan Africa to derive and internally validate a Universal Vital Assessment (UVA) score to predict in-hospital mortality in Africa [7]. We found that the UVA score had an overall area under the receiver operating characteristic curve (AUC) of 0.77 for all hospitalized patients and 0.75 for patients with known or suspected infection, which outperformed both qSOFA and the modified early warning score (MEWS) for all patients and in a large subset of patients with acute infection.

This finding was recently independently replicated in patients from Gabon where the UVA score also outperformed qSOFA in the prediction of in-hospital mortality [8]. Adaptation of the qSOFA score using UVA score cut-offs to create an amalgamated qSOFA score improved qSOFA performance in these patients but did not in an external validation cohort from Malawi [8]. These data suggest that different population characteristics such as HIV prevalence and case fatality rate may influence the performance of mortality risk scores, including the UVA score. A better understanding of a patient’s severity of illness at admission could allow for improved risk stratification in clinical studies and inform clinical decision-making including patient triage and allocation of resources. Therefore, the objectives of this study were to 1) externally validate the performance of the UVA score using previously collected data from patients hospitalized with acute infection in Rwanda; 2) determine whether changes in UVA score over time could predict outcomes; and 3) compare the performance of the UVA score to those of qSOFA and MEWS.

## Materials and methods

### Study participants

We performed a secondary analysis of data collected from adults ≥18 years admitted with an acute infectious disease to Gitwe District Hospital from the adult and pediatric Emergency Department of Gitwe District Hospital as well as eight referring health centers in the Ruhango District, Southern Province, Rwanda from March 2016 until March 2017 [9]. In the original study, using a data collection tool (S1 Fig), acute infection was defined as a suspected or confirmed infection, which was based on a clinical assessment and present for less than two weeks.

### Analyses

We summarized patient characteristics as frequency with percentage for categorical variables and median with interquartile range (IQR) for continuous variables. We used the Chi-square test for comparisons of proportions and the Mann-Whitney U test for comparisons of continuous variables. We calculated the UVA score for each participant from the time of admission and from 72 hours after admission. The UVA score includes points for temperature, heart rate, respiratory rate, systolic blood pressure, oxygen saturation, GCS score, and HIV serostatus [7]. Oxygen saturation was not measured in this patient cohort. Except for sensitivity analyses in which we imputed a two for missing HIV or oxygen saturation UVA score criteria, we did not impute missing values. We calculated the admission qSOFA score and the modified early warning score (MEWS) for each patient [2,10]. We calculated amalgamated qSOFA scores by inserting UVA score cut-offs for respiratory rate and systolic blood pressure into the qSOFA score, and modified UVA scores by removing the HIV criterion from the UVA score [8]. A comparison of the mortality risk score components and their associated point values are found in Table 1.

**Table 1:** A comparison of clinical mortality risk score components and their associated point values.

	UVA		Modified UVA		qSOFA		Amalgamated qSOFA		MEWS	
	Value	Points	Value	Points	Value	Points	Value	Points	Value	Points
GCS score	15	0	15	0	15	0	15	0	A (GCS 15)	0
	<15	4	<15	4	<15	1	<15	1	V (GCS 13)	1
	---	---	---	---	---	---	---	---	P (GCS 8)	2
	---	---	---	---	---	---	---	---	U (GCS 6)	3
SBP, mmHg	≥90	0	≥90	0	>100	0	≥90	0	≤70	3
	<90	1	<90	1	≤100	1	<90	1	71–80	2
	---	---	---	---	---	---	---	---	81–100	1
	---	---	---	---	---	---	---	---	101–199	0
	---	---	---	---	---	---	---	---	≥200	2
Respiratory Rate, brpm	<30	0	<30	0	<22	0	<30	0	<9	2
	≥30	1	≥30	1	≥22	1	≥30	1	9–14	0
	---	---	---	---	---	---	---	---	15–20	1
	---	---	---	---	---	---	---	---	21–29	2
	---	---	---	---	---	---	---	---	≥30	3
Heart rate, bpm	<120	0	<120	0	---	---	---	---	<40	2
	≥120	1	≥120	1	---	---	---	---	41–50	1
	---	---	---	---	---	---	---	---	51–100	0
	---	---	---	---	---	---	---	---	101–110	1
	---	---	---	---	---	---	---	---	111–129	2
	---	---	---	---	---	---	---	---	≥130	3
Temperature,°C	≥36	0	≥36	0	---	---	---	---	<35	2
	<36	2	<36	2	---	---	---	---	35–38.4	0
	---	---	---	---	---	---	---	---	≥38.5	2
Oxygen saturation, %	≥92	0	≥92	0	---	---	---	---	---	---
	<92	2	<92	2	---	---	---	---	---	---
HIV serostatus	Positive	2	---	---	---	---	---	---	---	---
	Negative/unknown	0	---	---	---	---	---	---	---	---

GCS, Glasgow coma scale; A, alert, V, verbal, P, pain, U, unresponsive; SBP, systolic blood pressure; brpm, breaths per minute; bpm, beats per minute.

We calculated the AUC for each score and compared them using the DeLong test for two correlated receiver operator characteristic curves [11]. Using commonly used cut-offs we calculated sensitivity, specificity, positive predictive value, and negative predictive value for in-hospital mortality for each score. We tested the differences between the UVA score cut-offs in their associations with mortality using multiple logistic regressions with simultaneous tests for general linear hypotheses, which included a statistical penalty for multiple comparisons using Tukey contrasts. We also determined the association between the 72 hour delta UVA score (72 hour UVA score minus admission UVA score) and in-hospital mortality.

### Ethical considerations

Approval for the original study and subsequent previously unplanned analyses was obtained from the National Health and Research Committee as well as the Rwanda National Ethics Committee (No.007/RNEC/2016). The study was conducted in accordance with ethical principles of the Declaration of Helsinki. Consent forms were translated into Kinyarwanda and written informed consent was obtained from all study participants before study enrollment. Our secondary analysis was not included in the original study protocol. We adhered to the Strengthening and Reporting of Observational Studies in Epidemiology (STROBE) guideline [12].

## Results

**Fig 1:**
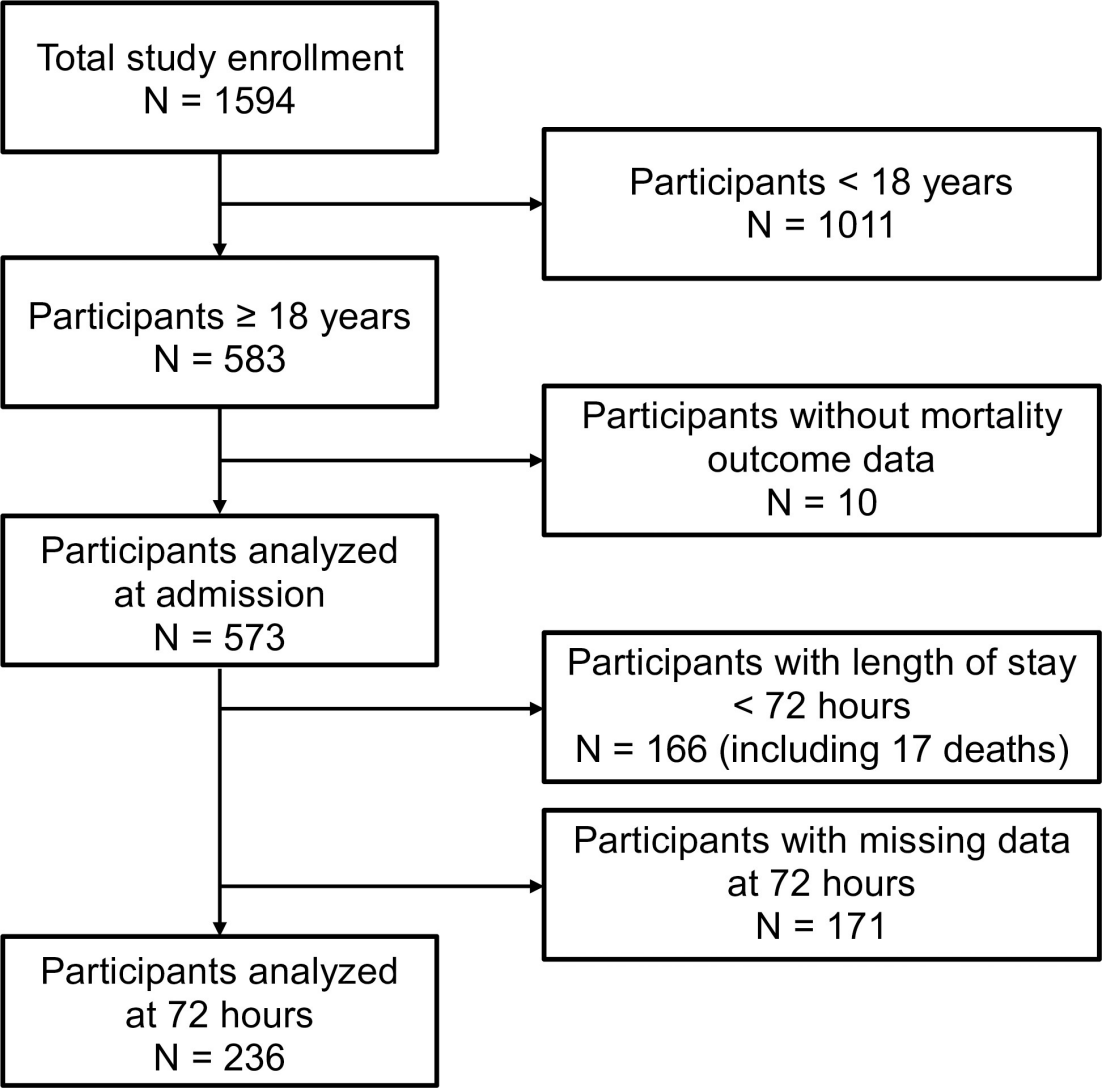
Flow diagram of participant inclusion in the external validation study of the UVA score in a cohort of patients admitted to hospital with acute infection in Gitwe, Rwanda.

**Fig 2:**
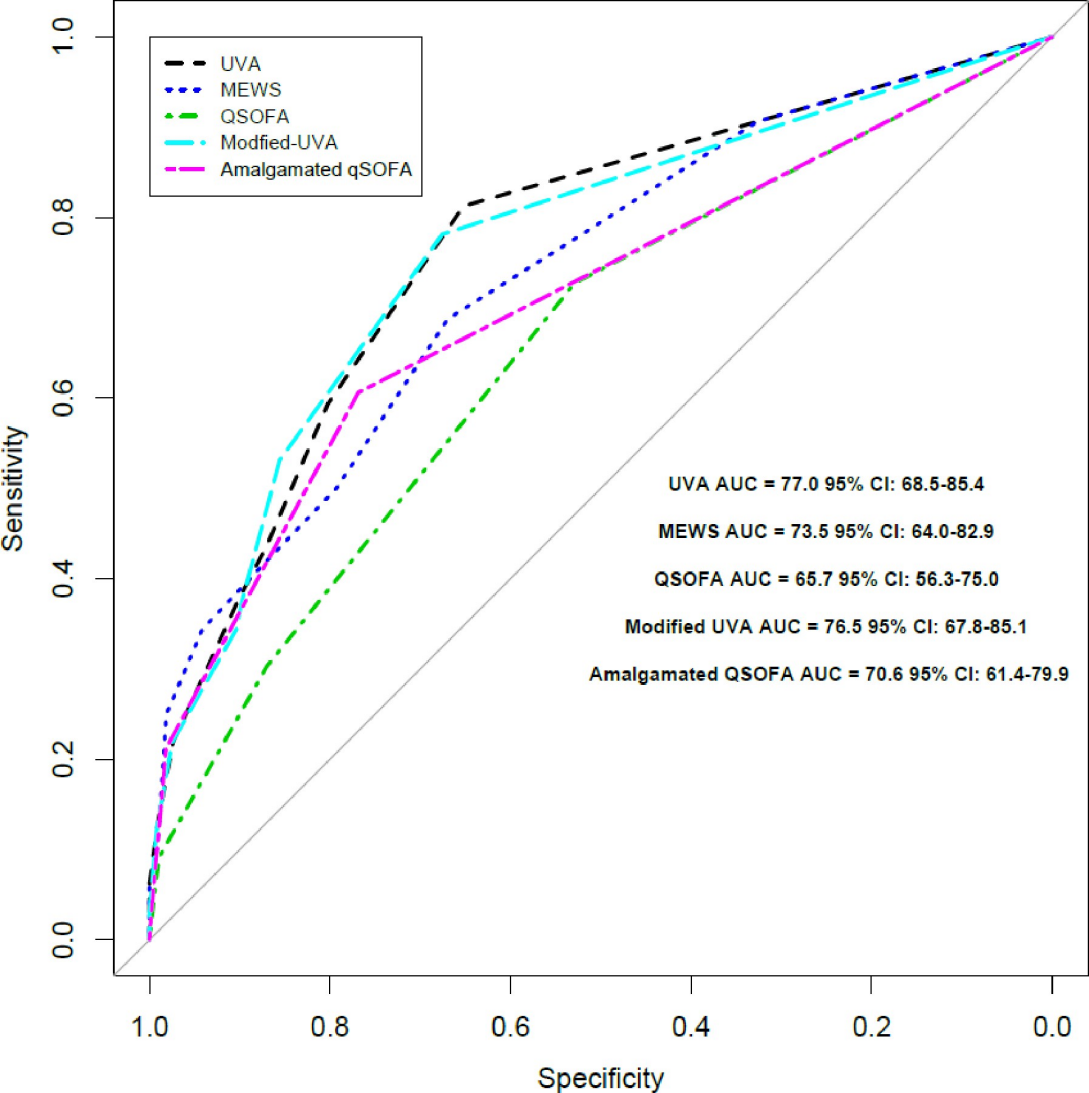
Receiver operating characteristic curves for different mortality risk scores used to predict mortality in adult patients admitted to hospital with acute infection in Gitwe, Rwanda.

We included data from 573 adult patients with known in-hospital mortality outcomes (Fig 1). In patients with available data, 358 of 566 (63%) were women, the median (IQR) age was 38 (28–58) years and 30 of 152 (20%) with a known HIV serostatus were living with HIV (Table 2). Death occurred in 40 of 573 (7%) patients. The admission AUCs (95% confidence interval [CI]) for the prediction of mortality by the scores were: UVA, 0.77 (0.68–0.85); modified UVA, 0.77 (0.68–0.85); qSOFA, 0.66 (0.56–0.75), amalgamated qSOFA, 0.71 (0.61–0.80); and MEWS, 0.74 (0.64–0.83) (Fig 2). There was a statistically significant difference between the AUC for UVA and qSOFA (p = 0.02) but not for UVA and MEWS (p = 0.3).

**Table 2:** Population characteristics of adult patients admitted to hospital with acute infection in Gitwe, Rwanda including a comparison between those who survived hospitalization and those who died in-hospital.

Variable	Missing n (%)	Total (n = 573)	Survived (n = 533)	Died in- hospital (n = 40)	p-value
Age (years), median (IQR)	0 (0)	38 (28–58)	37 (27–56)	62 (42–72)	<0.001
Female, n (%)	7 (1)	358 (63)	343 (65)	15 (38)	0.001
Living with HIV, n (%)	421 (89)	30 (20)	26 (19)	4 (31)	0.30
Temperature (°C), median (IQR)	13 (2)	37 (36–38)	37 (36–38)	37 (37–39)	0.27
Heart rate (bpm), median (IQR)	16 (3)	93 (80–109)	93 (79–108)	107 (87–130)	<0.001
Respiratory rate (brpm), median (IQR)	14 (2)	20 (19–20)	20 (19–20)	20 (19–22)	0.55
SBP (mmHg), median (IQR)	45 (8)	110 (97–122)	100 (98–122)	100 (84–110)	0.001
Oxygen saturation, median (IQR)	573 (100)	---	---	---	---
GCS score <15, n (%)	6 (1)	59 (10)	47 (9)	12 (30)	<0.001

Bpm, beats per minute; brpm, breaths per minute; SBP, systolic blood pressure; GCS, Glasgow coma scale.

Note that the percentages shown for the categories of total, survived, and died in-hospital are calculated from the non-missing values for each variable.

In a sensitivity analysis of patients with complete data, we found that the AUCs (95% CI) for the prediction of mortality by the scores were: UVA (n = 492), 0.77 (0.68–0.85); modified UVA (n = 510), 0.76 (0.68–0.85); qSOFA (n = 510), 0.66 (0.57–0.76); amalgamated qSOFA (n = 510), 0.72 (0.62–0.81); and MEWS (n = 510), 0.73 (0.64–0.83). We conducted additional sensitivity analyses whereby we imputed a two for oxygen saturation and determined the upper and lower UVA score AUC bounds (95% CI) as 0.77 (0.69–0.86) and 0.77 (0.69–0.86), respectively. Similarly, we also imputed a two for HIV and established the upper and lower UVA score AUC bounds (95% CI) as 0.77 (0.69–0.86) and 0.75 (0.65–0.85), respectively.

**Fig 3:**
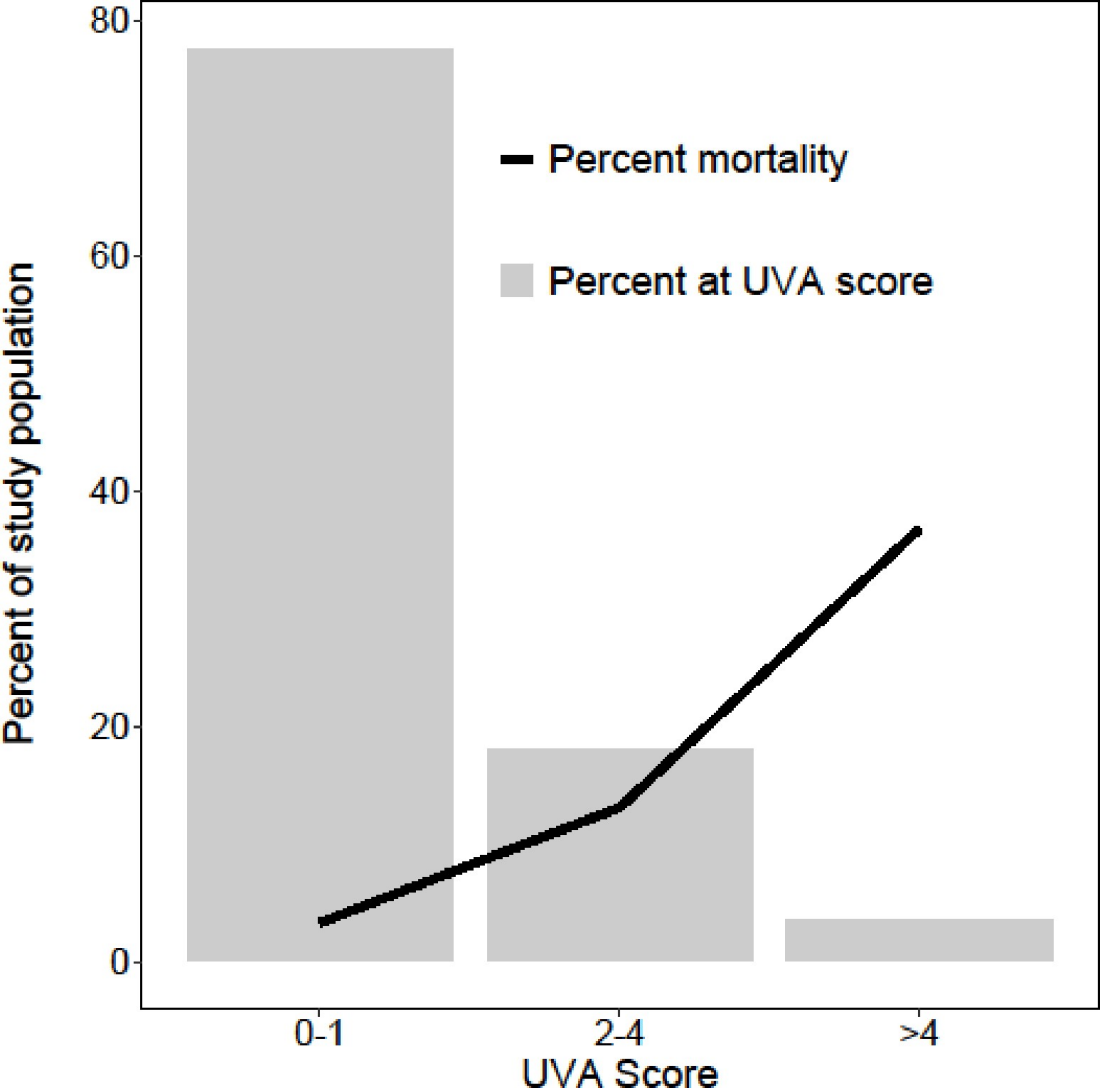
The frequency and associated case fatality rate of UVA score risk categories (low 0–1, medium 2–4, high >4) of adult patients admitted to hospital with acute infection in Gitwe, Rwanda.

**Fig 4:**
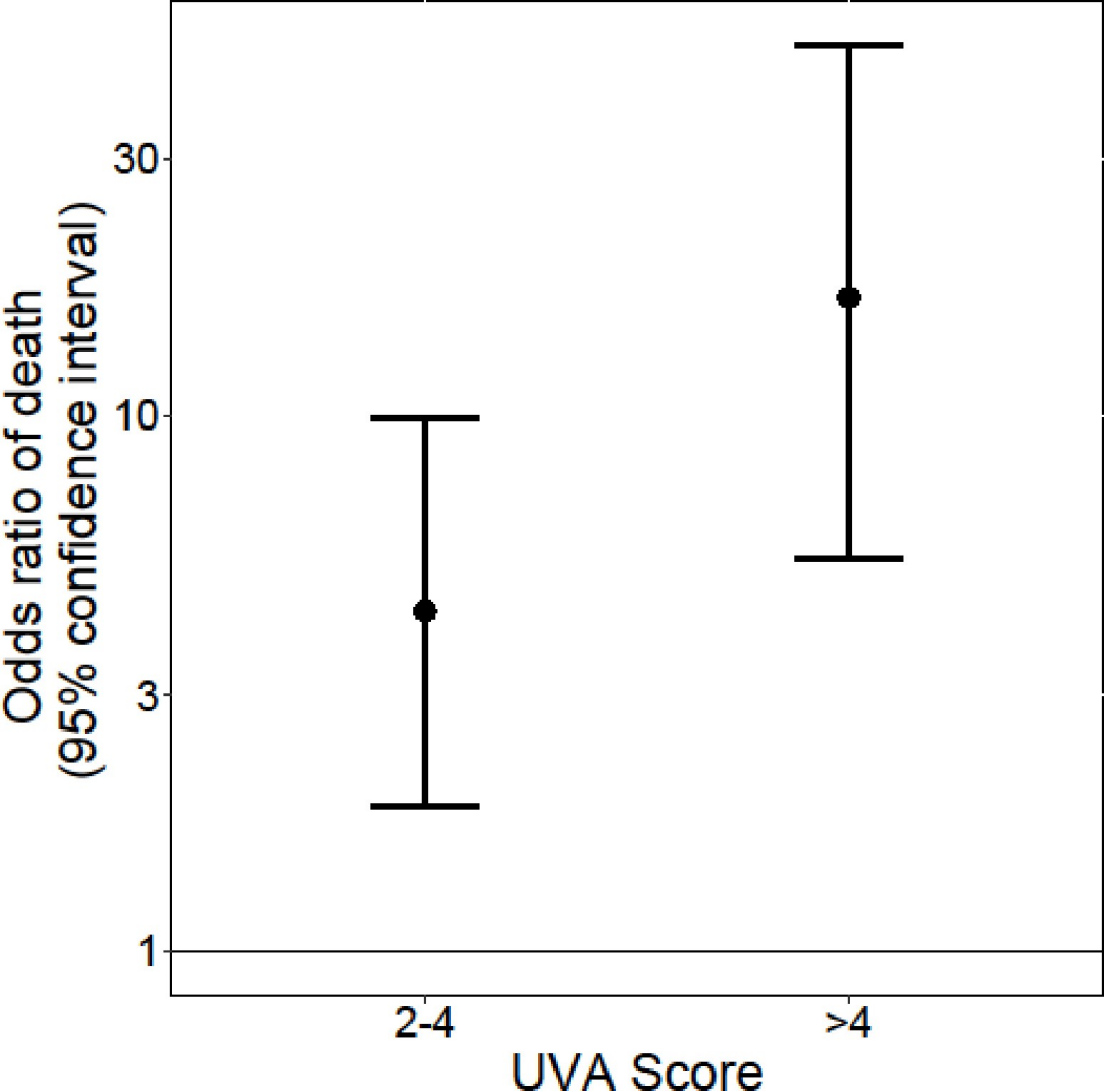
Odds ratios with 95% confidence intervals for in-hospital mortality associated with medium (2–4) and high (>4) risk UVA score categories compared to the low (0–1) risk UVA score category for adult patients admitted to hospital with acute infection in Gitwe, Rwanda.

Increasing UVA scores were associated with increased case fatality rates and odds ratio of death (Figs 3 and 4; Table 3). The positive predictive values (95% CI) of the scores at commonly used cut-offs were: UVA >4, 0.35 (0.15–0.59); modified UVA >4, 0.35 (0.15–0.59); qSOFA >1, 0.14 (0.07–0.24); amalgamated qSOFA >1, 0.44 (0.20–0.70); and MEWS >5, 0.14 (0.08–0.22) (Table 4). When calculated using data obtained from patients at 72 hours (N = 236), the AUC (95% CI) for the prediction of mortality by UVA was 0.59 (0.43–0.74). The Chi-Square test for linear trend did not identify an association between mortality and delta UVA score (p = 0.82).

**Table 3:** A multivariable analysis of the differences between the UVA cut-offs in their associations with mortality.

Comparator score cut-offs		Odds ratio	95% confidence interval	P value
UVA 2–4	UVA 0–1	4.31	1.9–9.8	0.0015
UVA >4	UVA 0–1	16.56	5.6–48.9	<0.001
UVA >4	UVA 2–4	3.84	1.3–11.7	0.046

**Table 4:** A comparison of the sensitivity, specificity, positive predictive value, and negative predictive value including 95% confidence intervals in parentheses for in-hospital mortality for adult patients admitted to hospital with acute infection in Gitwe, Rwanda according to different cut-offs for the UVA score, Modified UVA score, qSOFA score, Amalgamated qSOFA score, and MEWS.

Score	Sensitivity	Specificity	Positive predictive value	Negative predictive value
UVA, low risk (0–1)	0.47 (0.32–0.64)	0.19 (0.16–0.22)	0.04 (0.03–0.06)	0.83 (0.75–0.89)
UVA, medium risk (2–4)	0.35 (0.21–0.52)	0.84 (0.80–0.87)	0.14 (0.08–0.22)	0.94 (0.92–0.96)
UVA, high risk (>4)	0.17 (0.07–0.33)	0.98 (0.96–0.99)	0.35 (0.15–0.59)	0.94 (0.92–0.96)
Modified UVA, low risk (0–1)	0.53 (0.36–0.68)	0.14 (0.11–0.17)	0.04 (0.03–0.07)	0.80 (0.70–0.87)
Modified UVA, medium risk (2–4)	0.30 (0.17–0.47)	0.89 (0.86–0.91)	0.16 (0.09–0.27)	0.94 (0.92–0.96)
Modified UVA, high risk (>4)	0.17 (0.07–0.33)	0.98 (0.96–0.99)	0.35 (0.15–0.59)	0.94 (0.92–0.96)
qSOFA (>1)	0.30 (0.16–0.49)	0.87 (0.84–0.90)	0.14 (0.07–0.24)	0.95 (0.92–0.97)
Amalgamated qSOFA (>1)	0.17 (0.07–0.33)	0.98 (0.97–0.99)	0.44 (0.20–0.70)	0.94 (0.92–0.96)
MEWS (>5)	0.50 (0.32–0.68)	0.79 (0.75–0.83)	0.14 (0.08–0.22)	0.96 (0.94–0.98)

## Discussion

In this study, we externally validated the ability of the UVA score to predict in-hospital mortality in a cohort of patients with acute infection admitted to a hospital in Rwanda who were not part of the UVA score derivation cohort [7]. In doing so, despite a 10% difference in case fatality rates between the derivation and validation cohorts (17% vs 7%), we found that the AUC for the prediction of in-hospital mortality was identical between derivation and validation cohorts at 0.77 [7,9]. Increasing UVA scores were associated with increased case fatality rates and odds ratio of death.

In a separate study from Uganda, the AUC for the UVA score was 0.82 [13]. Similar to studies in Gabon and Malawi, we found that the UVA score outperformed qSOFA [8]. We also found that an amalgamated qSOFA score that used UVA cut-offs that were derived from a pooled African population improved the performance of qSOFA. In our study, using a cut-off score of >1, the amalgamated qSOFA had the highest positive predictive value (0.44) of all the tested scores. After successful prospective validation, both the UVA score and the amalgamated qSOFA could be used clinically to identify patients at risk for in-hospital death, which could be evaluated in a cluster randomized trial to determine whether identification of mortality risk leads to improved outcomes in different clinical settings. The UVA score has been used by others to control for severity of illness at admission in observational clinical studies and it could also be used for risk stratification in interventional studies [14,15]. For example, since steroid therapy for COVID-19 is based on severity of illness, the UVA score might be a useful indicator for steroid therapy in resource limited settings, which would need to be tested prospectively. Since the derivation of the Sepsis-3 definitions did not include any data from patients from LMICs, the UVA score, which has consistently outperformed qSOFA in different African populations, may be useful to help determine best definitions for sepsis in Africa [2,16].

A recent study by Klinger et al found a similar AUC for the UVA score (0.71) among patients with suspected infection who were admitted to a tertiary hospital in Rwanda, which was not used to conduct our study [6]. Although numerically larger, the AUC for UVA was not statistically superior to that of qSOFA (0.65) or MEWS (0.69). However, this may be because in the Klinger study the UVA score was calculated at the time of suspected infection, which was frequently not at the time of admission. Accordingly, there may have been a survivor bias in the results of the Klinger study analysis of the scores as participants who died rapidly after admission to the hospital before they could be screened, or who died before infection was suspected, were not included in the analyses. Additionally, almost half of the study population were surgical patients, a patient population that was not included in our original UVA score derivation study. Despite these differences in study design and populations, the UVA score maintained good performance in the Klinger study. At a cut-off of 4, the positive predictive value of the UVA score (0.41) was superior to both qSOFA (0.31) and MEWS (0.36) at cut-offs of 2 and 4, respectively. It is possible that the calculation of the UVA score at admission and in a more similar study population would have provided improved mortality prediction, but this cannot be known from the available data.

Similar to the findings of the Klinger study, we also found that the UVA score calculated at 72 hours after admission performed less well than when calculated at admission. This difference in performance may be because improvement in vital signs in the short term do not necessarily predict improved outcomes in the long term due in part to immunodeficiency, poor nutrition, and a lack of appropriate antimicrobial therapy [17–19]. For example, in a study of the resuscitation of patients with sepsis in Uganda, we found that vital signs and whole blood lactate improved over the first 6 hours but that these improvements were not associated with improved case fatality rates [18]. Rather, mortality was associated with unmodifiable risk factors such as mid-upper arm circumference, a proxy for nutritional status, and low Glasgow coma scale score, which is not typically quickly reversed in critically ill patients.

Furthermore, in a study of septic patients in Uganda, we found that increased vital sign acquisition alone was associated with worse outcomes, perhaps reflecting increased clinical concern for poor outcomes [17]. Therefore, the subset of patients with vital signs available at 72 hours to calculate the UVA score in the current study may have been more critically ill and not representative of outcomes for all patients at 72 hours. In another study from Uganda, we found that 61% of deaths that occurred in patients admitted with sepsis occurred in the first 4 days of admission [19]. Therefore, the ability to survive to 72 hours may be more predictive of subsequent survival than changes in vital signs over the first few days of admission [19]. It is not known whether a change in UVA score, or vital signs in general, between admission and 6 to 72 hours is predictive of outcomes in this setting.

Additionally, early changes in vital sign trajectories may not predict mortality due to inappropriate antimicrobial therapy. In high HIV and tuberculosis (TB) prevalent settings such as East Africa, including Rwanda, where in a recent study of hospitalized patients with sepsis 35% were living with HIV, TB is the leading cause of bloodstream infections in septic patients and is associated with high case fatality rates of approximately 20–50% [20–22]. In a pooled analysis of limited post-mortem studies of hospitalized patients from sub-Saharan Africa with HIV and a previously unknown cause of death, the estimated prevalence of TB was 40% [23]. Among them, TB was disseminated in 88% and the cause of death in 91%. Despite the high prevalence and burden of TB in septic patients, TB can be difficult to diagnose clinically. Mycobacterial blood cultures are not usually available to clinicians and have a long turnaround time. Point-of-care diagnostics such at Gene Xpert MTB/RIF and urinary lipoarabinomannan have limited sensitivity [24]. Therefore, even if a transient improvement in UVA score, or vital signs in general, occurs with resuscitation over 72 hours, failure to treat underlying TB or other antimicrobial resistant pathogens would likely lead to death [19].

Our study has limitations. First, the population studied was from a single center in Rwanda with a relatively low HIV prevalence, which may not be representative of other African settings. However, the UVA score, which was derived from pooled data from studies of hospitalized patients in 6 different countries in sub-Saharan Africa, has now been validated in separate populations in Gabon, Uganda, and Rwanda with similar results, which provides confidence in its generalizability across sub-Saharan Africa. Second, this was a retrospective validation study of the UVA score and a prospective study could further determine how the UVA score can be best deployed in different clinical settings. Third, our data were limited by the absence of oxygen saturation data. Nonetheless, the UVA score still performed well and might be improved with this additional information when it is available to clinicians. Similarly, the modified UVA score without HIV status input retained good predictive ability suggesting robustness in a real world setting where clinical data including oxygen saturation and HIV serostatus are often not available. In these cases, the amalgamated qSOFA score may offer additional predictive information. Finally, we did not have complete data at 72 hours which may not have been missing at random due to early deaths, so our assessment of the utility of delta UVA score at that time point was not conclusive. Further studies could evaluate whether the delta UVA score at earlier time points provides additional predictive information regarding mortality.

## Conclusions

We found that the admission UVA score had good predictive ability for mortality in adult patients admitted to hospital with suspected or confirmed acute infection in Rwanda. Increasing UVA scores were associated with increased case fatality rates and odds ratio of death. Sensitivity analyses revealed robust performance of the UVA score even when oxygen saturation and HIV serostatus were missing. The delta UVA score at 72 hours was not predictive of mortality but this analysis was limited by a lack of available data. After successful prospective validation, the UVA score could be used for clinical application to assist with triage decisions and clinical interventions. It may also be used for baseline risk stratification in clinical studies and applied to a clinical definition of sepsis in Africa.

## Supporting information

S1 Fig Data collection tool used in the original study that generated data used in the external validation of the UVA score in Rwanda study. (TIF)
