# Supplementary figures and images for: Performance of the Universal Vital Assessment (UVA) mortality risk score in hospitalized adults with infection in Rwanda: A retrospective external validation study
Source: PLoS One. 2022 Mar 23;17(3):e0265713. doi: 10.1371/journal.pone.0265713 (PMC8942262; doi:10.1371/journal.pone.0265713)

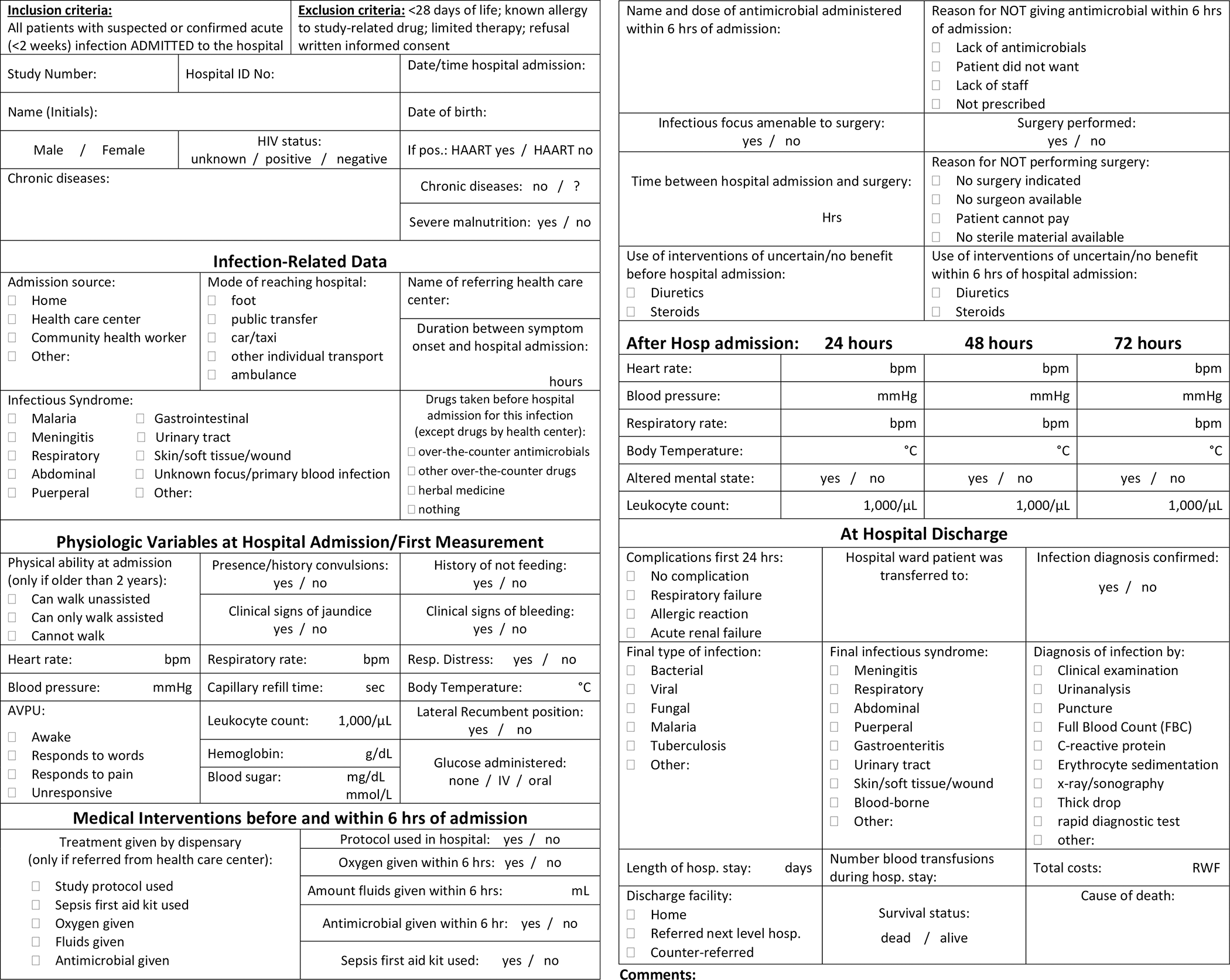

Supplement: S1 Fig — (TIF) [file pone.0265713.s001.tif]
